# Electroencephalographic characterization of subgroups of children with learning disorders

**DOI:** 10.1371/journal.pone.0179556

**Published:** 2017-07-14

**Authors:** Milene Roca-Stappung, Thalía Fernández, Jorge Bosch-Bayard, Thalía Harmony, Josefina Ricardo-Garcell

**Affiliations:** Departamento de Neurobiología Conductual y Cognitiva, Instituto de Neurobiología, Universidad Nacional Autónoma de México, Querétaro, México; Centro de Neurociencias de Cuba, CUBA

## Abstract

Electroencephalographic alterations have been reported in subjects with learning disorders, but there is no consensus on what characterizes their electroencephalogram findings. Our objective was to determine if there were subgroups within a group of scholars with not otherwise specified learning disorders and if they had specific electroencephalographic patterns. Eighty-five subjects (31 female, 8–11 years) who scored low in at least two subscales -reading, writing and arithmetic- of the Infant Neuropsychological Evaluation were included. Electroencephalograms were recorded in 19 leads during rest with eyes closed; absolute power was obtained every 0.39 Hz. Three subgroups were formed according to children’s performance: Group 1 (G1, higher scores than Group 2 in reading speed and reading and writing accuracy), Group 2 (G2, better performance than G1 in composition) and Group 3 (G3, lower scores than Groups 1 and 2 in the three subscales). G3 had higher absolute power in frequencies in the delta and theta range at left frontotemporal sites than G1 and G2. G2 had higher absolute power within alpha frequencies than G3 and G1 at the left occipital site. G3 had higher absolute power in frequencies in the beta range than G1 in parietotemporal areas and than G2 in left frontopolar and temporal sites. G1 had higher absolute power within beta frequencies than G2 in the left frontopolar site. G3 had lower gamma absolute power values than the other groups in the left hemisphere, and gamma activity was higher in G1 than in G2 in frontopolar and temporal areas. This group of children with learning disorders is very heterogeneous. Three subgroups were found with different cognitive profiles, as well as a different electroencephalographic pattern. It is important to consider these differences when planning interventions for children with learning disorders.

## Introduction

Learning disorders (LD) are developmental disorders that affect the brain’s ability to efficiently and accurately perceive or process verbal or non-verbal information, and there are subsequent difficulties learning key academic skills. According to the American Psychiatric Association [1], LD are diagnosed when an individual’s achievement in individually administered, standardized tests in reading, mathematics and/or written expression is substantially below that expected for their particular age, schooling, and level of intelligence. Learning disorders can be classified as specific (reading disorder, mathematic disorder, or disorder of written expression) or not otherwise specified (LDNOS) [1]. There are diverse estimates on the incidence of LD that range from 5% to 15% [1,2,3]. Because LDNOS can be diagnosed in one or many distinct domains, it is a heterogeneous group and a challenging task to describe it.

In numerous studies, the resting state electroencephalogram (EEG) with eyes closed has been recorded in subjects with learning disorders with the objective of observing an electroencephalographic pattern that discriminates between this group and subjects with good academic performance [4,5,6,7,8,9,10,11,12,13,14,15,16,17,18,19]. Although there is no consensus yet about what characterizes the EEG of learning disabled children, the most commonly reported pattern is an excess of slow activity, mainly in the theta frequency range [4,8,9,10,13,15,16,18,20], and an alpha activity deficit [6,7,8,10,11,15,16] when compared to typical children. Few of these studies included subjects with disability in more than one academic skill [4,7,10,16,19,20]. Excess delta activity has been reported when severe disability is observed [13]. It is clear from the reported electroencephalographic alterations in this group that brain function is altered, but the observed alterations are not specific.

LDNOS is the most frequent of LDs, the majority of children with LD present alterations in two or more cognitive domains. The Diagnostic and Statistical Manual of Mental Disorders IV [1] states that mathematics disorder and disorder of written expression most commonly occur in combination with reading disorder. Due to the significant heterogeneity of subjects with learning disorders [21,22], the authors consider it necessary to explore a) if there are subgroups within this group and b) if these subgroups exhibit specific electroencephalographic patterns.

Subtypes in a group of children with LD have been defined on the basis of the patterns of deficits in their cognitive abilities, and differences have been found between them in terms of their EEG activity in event related potentials [23,24,25]. Jäncke and Alahmadi [20] explored differences in the resting state EEG using an independent component analysis, between healthy control children, children with verbal disabilities, and children with LDNOS; they found differences in EEG oscillations in an eyes open condition in the left temporal cortex in delta, theta, alpha and beta-1 frequencies as well as in alpha in an eyes closed condition in the mesial paracentral cortex extending into the superior parietal lobe. They further found a higher theta/alpha ratio in the frontal regions in children with LDNOS.

Clarke *et al*. [26] found three subtypes of children with the combined type of attention-deficit/hyperactivity disorder according to their EEG profiles. To the best of our knowledge, subtypes within a group of children with LDNOS have not been defined considering electrical activity. The aim of this study is to identify the resting state EEG similarities and differences within a group of scholars with LDNOS to determine if there are subgroups that can be defined according to problematic domains and EEG patterns. While Clarke *et al*. [26] explored clusters formed from EEG data, in this study we found three groups of children with LDNOS according to their cognitive performance and explored if there were differences between them in their brain electrical activity.

## Methods

Eighty-five right-handed subjects (31 female) between 8 and 11 years of age (9.2 ± 0.96) were included in this study. The children were volunteers from elementary schools, who were referred by their teachers due to problems in academic performance. The mothers of the selected children had completed at least elementary education, and their family income was at least 50% of the minimum wage per subject to avoid major cultural disadvantages. The children did not have psychiatric disorders beyond their LDs (M.I.N.I.-KID [27]), and their neurologic examinations were normal. Although LD children often have some deficit in attentional processes, children in our study did not satisfy the criteria to be classified as Attention Deficit Hyperactivity Disorder, and no one was hyperactive. Subjects were assessed with the Wechsler Intelligence Scale for Children–IV (WISC-IV [28]), and the Child Neuropsychological Assessment (Evaluación Neuropsicológica Infantil, ENI), which was developed by Matute, Rosselli, Ardila and Ostrosky-Solis [29] with Mexican Norms. All children had a Full Scale Intelligence Quotient (FSIQ) score of 70 or higher to exclude mental retardation. The following three domains of the ENI were evaluated: reading, writing, and arithmetic, and the following three variables were evaluated for each domain: reading (accuracy, comprehension, and speed); writing (accuracy, narrative composition, and speed); and counting, numeric management and calculation. Raw scores are transformed to percentiles, according to the norms of the ENI (from Mexican population). A percentile of 26 or higher is classified as average, a percentile from 11 to 25 is classified as lower average, low scores are percentiles from 3 to 10, and a percentile of 2 or lower is classified as extremely low. All subjects scored low in at least 1 domain and low average in at least another domain (reading, writing and/or arithmetic). A direct variable k-means cluster analysis was performed using the Ward method with the nine variables that were evaluated with the ENI to identify subgroups within this group of children with LDNOS.

EEG was recorded during rest with eyes closed using a Medicid^™^ IV system (*Neuronic Mexicana*, *S*.*A*.; Mexico) and Track Walker TM v5.0 data system, from the 19 leads of the 10–20 system (ElectroCap^™^, International Inc.; Eaton, Ohio) referenced to the linked earlobes (A1–A2). The amplifier bandwidth was set between 0.5 and 50 Hz. All electrode impedances were at or below 10 kΩ, and the signal was amplified with a gain of 20,000. EEG data were sampled every 5 ms and edited off-line. On average, 24 artefact-free segments of 2.56 s were used to analyse the recordings. The data were fast Fourier transformed to obtain cross-spectral matrices, and the absolute power (AP) with geometric power correction [30], which accounts for 42% of the variability that is not related to physiological factors, was calculated every 0.39 Hz. A one-way ANOVA test was performed to compare the EEG AP from the three groups. No significant differences were found between the three groups in the age; for this reason, raw AP values with geometric power correction were used. The significance threshold was corrected to account for multiple comparisons using a randomization test.

The Ethics Committee of the Neurobiology Institute of the National Autonomous University of Mexico approved this study, which followed the Ethical Principles for Medical Research Involving Human Subjects established by the Declaration of Helsinki. Informed consent was signed by all children and their parents.

## Results

One hundred and ninety-three elementary students were referred by their teachers due to academic problems. Of these children, 46% were excluded for several reasons: 6% had a FSIQ of 70 or lower (which is classified as mental retardation), 6% presented a specific learning disorder (not LDNOS), 6% were diagnosed with Attention Deficit Disorder, 9% presented psychiatric disorders beyond their LDNOS (depression, anxiety, autism, disruptive), 4% presented neurological problems, 4% had a very low family income and their caregivers did not complete elementary education, another 4% had language problems, 3% were left-handed, and 14% did not complete their evaluations.

A visual inspection of the resulting dendrogram from cluster analysis revealed three independent clusters of children with different cognitive characteristics. Descriptive measures for the three groups are presented in Table 1.

**Table 1:** Descriptive measures of the three groups obtained from the cluster analysis.

Groups	n	FSIQ	Age
**1**	**24**	**90.91(±8.97)**	**9.33(±0.81)**
**2**	**26**	**90.81(±9.58)**	**9.17(±0.96)**
**3**	**35**	**83.23(±10.36)**	**9.14(±1.07)**

FSIQ: Full Scale IQ score

The age and Full Scale Intelligence Quotient (FSIQ) of the three groups were compared with an ANOVA test, and the results are displayed in the two first rows of Table 2. No significant difference was found between groups in their ages. Groups 1 (p = 0.0017) and 2 (p = 0.0020) have significantly higher FSIQ scores than Group 3.

**Table 2:** Results of the ANOVA test and Post hoc analysis performed with age, FSIQ, and reading, writing, and arithmetic variables from the Escala Neuropsicológica Infantil (ENI), comparing the three groups.

Variable		ANOVA		Post hoc					
		GlobalF(2,81)	p	Group1>Group2		Group1>Group3		Group2>Group3	
				t(47)	p	t(56)	p	t(59)	p
Age		1.28	.2848	-.97	.1676	-1.66	.051	-.57	.2847
FSIQ		6.24	.0030	.04	.4839	3.06	.0017	3.00	.0020
Reading	Accuracy	17.64	1.0e-5	3.42	.0007	5.53	1.0e-5	1.92	.0301
	Comprehension	4.30	.0167	1.64	.0537	2.65	.0053	1.30	.0985
	Speed	18.88	1.0e-5	4.28	1.0e-5	5.21	1.0e-5	1.01	.1573
Writing	Accuracy	104.9	1.0e-5	11.09	1.0e-5	15.29	1.0e-5	1.43	.0786
	Composition	48.78	1.0e-5	-3.78	.0002	3.99	.0001	12.83	1.0e-5
	Speed	.12	.8886	.11	.4555	.46	.3237	.36	.3601
Arithmetic	Counting	.43	.6502	-.93	.1780	-.60	.2754	.41	.3416
	Numeric management	8.28	.0005	.67	.2522	3.57	.0004	3.25	.0010
	Calculation	13.89	1.0e-5	.15	.4405	4.09	.0001	4.95	1.0e-5

FSIQ: Full Scale IQ score

**Fig 1:**
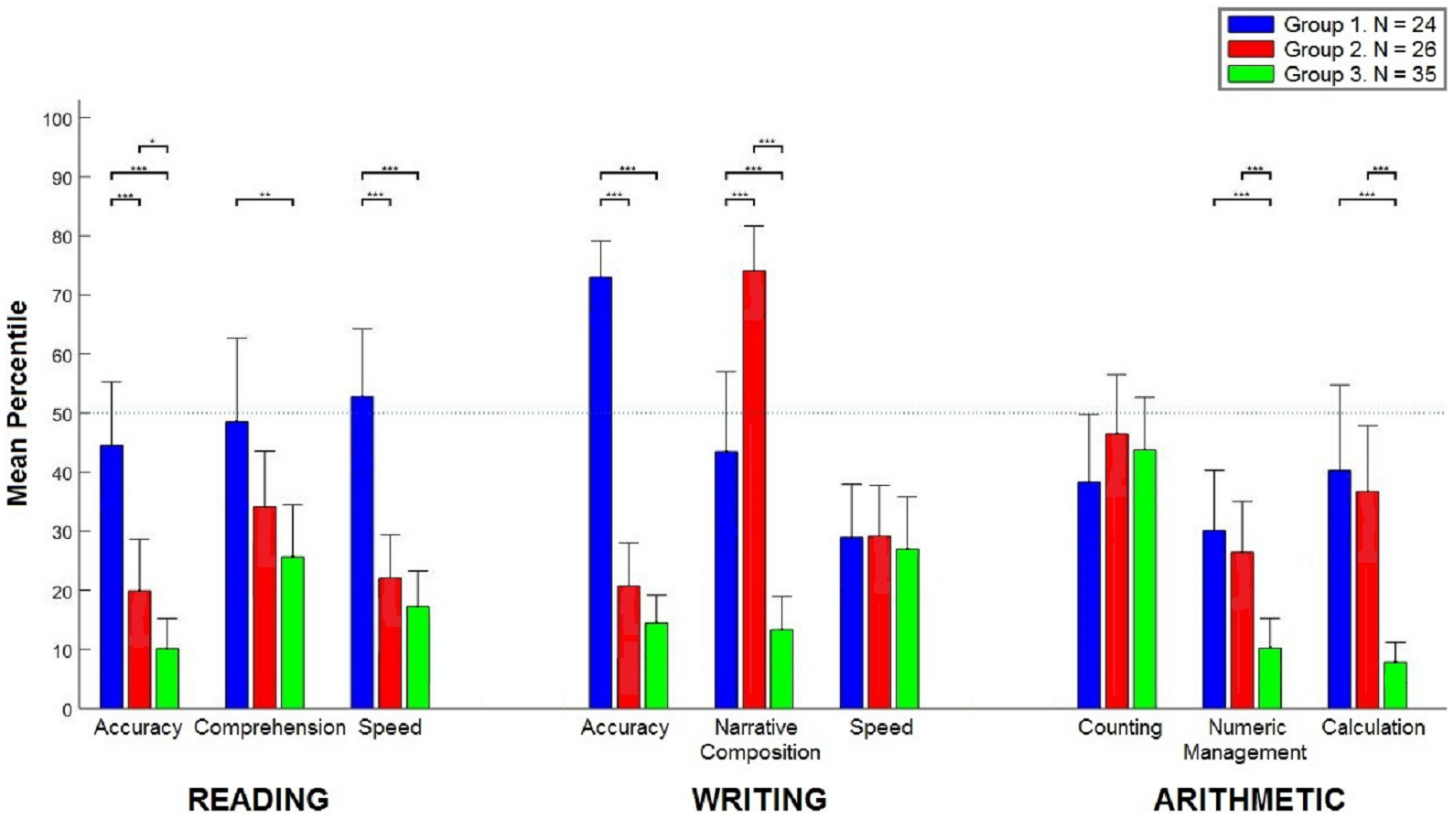
Mean percentiles of Groups 1, 2 and 3 in each of the subtests for the reading, writing and arithmetic domains of the Escala Neuropsicológica Infantil (ENI). Almost all the mean percentiles reached by the groups fall below the 50^th^ percentile (highlighted with a horizontal line). Significant differences are identified at the top (* for p<0.05, ** for p<0.01 and *** for p<0.001).

The results of the ANOVA test that was performed with ENI variables comparing the three groups are also displayed in Table 2. Group 1 had significantly (p<0.05) higher scores than Group 2 in the reading speed (p = 1.0e-5) and reading (p = 0.0007) and writing accuracy (p = 1.0e-5), and Group 2 had significantly better performance than Group 1 in the composition (p = 0.0002). Group 3 had significantly lower scores than Group 1 in the three reading domains (p = 1.0e-5, p = 0.0053, and p = 1.0e-5) as well as in writing accuracy (p = 1.0e-5), composition (p = 0.0001), numeric management (p = 0.0004) and calculation (p = 0.0001). Group 3 also had significantly lower scores than Group 2 in reading accuracy (p = 0.0301), writing composition (p = 1.0e-5), numeric management (p = 0.001), and calculation (p = 1.0e-5) (Fig 1).

**Fig 2:**
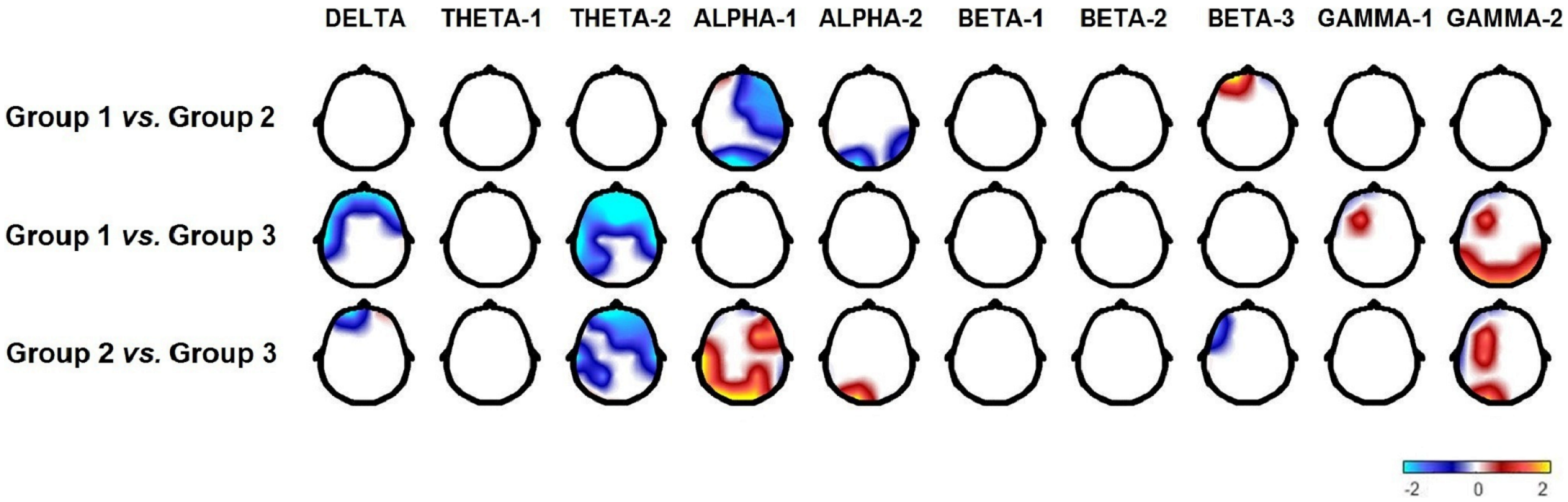
Topographic maps of the t values that represent the differences between groups in each broadband EEG (delta, theta-1, theta-2, alpha-1, alpha-2, beta-1, beta-2, beta3, gamma-1 and gamma-2). Note that Groups 1 and 2 have less slow activity and more gamma activity than the Group 3; in addition, Group 2 has more alpha activity and less beta-3 activity than Group 3. Comparisons between Groups 1 and 2 show that Group 1 has less alpha activity and more beta-3 activity than Group 2. The localization of these differences is discussed in relation to the tasks associated with the corresponding area.

A narrow band analysis was performed to explore the differences in AP values between groups; however, topographic maps of the differences between groups are shown in each broadband (delta, theta-1, theta-2, alpha-1, alpha-2, beta-1, beta-2, beta3, gamma-1 and gamma-2) in order to better visualise the results (Fig 2). Group 3 has more frontotemporal activity in the delta range than Groups 1 and 2. Group 3 has more bilateral frontotemporal and left parietal and occipital AP in the theta range than Group 1. Group 3 has more frontal activity in the theta range, mostly left, and left parietal activity in the theta range than Group 2. Group 3 has less AP in the low alpha range in the occipital and posterior temporal leads, mostly in the left hemisphere, and it has less activity in the fast alpha range in the left occipital area than Group 2. Group 3 has more activity in the low beta range in the left parietal and anterior temporal areas than Group 1. Group 3 has more AP in the low beta range in the left frontopolar lead and more activity in the fast beta range in the frontopolar and anterior temporal areas than Group 2. Group 3 has less activity from 30 to 50 Hz in the left frontotemporal, occipital and bilateral posterior temporal areas than Group 1. Group 3 has less AP, ranging from 37 to 50 Hz, in the left frontal, bilateral occipital and posterior temporal areas than Group 2.

Group 2 has higher AP in 2.73 Hz in Fz than Group 1. Group 2 has more activity in the low and fast alpha ranges in the left occipital area. Group 2 also has more activity in the low alpha range in bilateral temporal, right frontal and central areas, and it has higher AP in the fast alpha range in the left frontal areas than Group 1. Group 1 has higher AP from 21 to 28 Hz in the frontopolar areas and C4, Pz, T6 and F3 leads than Group 2. Group 1 has higher AP, from 30 to 38 Hz, than Group 2 in the left frontopolar and posterior temporal areas.

## Discussion

The clinical criteria established in the Diagnostic and Statistical Manual of Mental Disorders-IV [1] was used in this study to diagnose children with LDNOS. Although all of the subjects belonged to the same clinical group, within it we found three distinct groups according to their performance. There are two main hypotheses about the atypical processing patterns underlying learning disorders [31]. The common deficit hypothesis states that all children with LD have problems in common processing patterns. Supporting this hypothesis, Swanson [32] proposes a common deficit in executive functions that essentially involve working memory [33,34]. Other authors considered that the deficit is specifically in the phonological loop and central executive, as proposed by Baddeley [31,35,36,37,38]. Hari and Renvall [39] hypothesized that difficulties in reading acquisition could be due to a sluggish shift in attention [40]. The domain-specific cognitive hypothesis proposes the existence of subgroups of children with LD who have specific deficits. Siegel [41] supports this hypothesis, postulating that there is evidence of subgroups of children with LD who have particular characteristics and conditions that consistently predict specific patterns of learning difficulties. Our findings support both hypotheses; the common deficit hypothesis is supported by the fact that the three groups have significantly low scores (below 50th percentile) in all tested domains. All groups scored lower than the 30th percentile in writing speed and numeric management. Although all children perform lower than the average, we found three groups of children that had particular cognitive deficits and distinctive electroencephalographic patterns (domain-specific cognitive hypothesis). Not only were the groups different regarding their reading, writing and arithmetic results, Group 3 had a significantly lower IQ score than the other two groups.

Because children with LD form a very heterogeneous group, there have been several attempts to characterize subgroups within this group. One was the study performed by Hale, Casey and Ricciardi [42] where the authors found three clusters based on core subtest scores of the WISC-IV of children with academic difficulties. There was a globally low cluster that differed significantly from the other two clusters on the Word Reading, Numerical Operations, and Spelling subtests of the Wechsler Individual Achievement Test-II, whereas the other two groups did not differ from one another in any of the subtests. Korhonen [43] studied a group of children with LD and found five groups using a cluster analysis; one was a group with general deficiencies. We also found a group (Group 3) with severe deficiencies in the three areas we tested with the Neuropsychological Evaluation, who also had a lower total IQ score than the other two groups. Subgroups can be found even in specific learning disabilities, such as dyslexia. Boder [44] described three subtypes of dyslexia: dysphonetic, dyseidetic, and mixed; these have also been found in the Italian language [45].

It is important to note that we recorded resting EEGs with eyes closed for this study; therefore, our discussion focuses on the features that are known to modify resting EEG rhythms. Age is probably the most influential of these features; numerous studies have reported its effect on EEG since Petersen and Olofsson’s descriptions in 1971 [46]. They all report that there is a decrease in the mean amplitude [47], absolute power [13], and relative power [48] in the delta and theta frequencies from childhood to adult age. The fact that children with learning disabilities have higher delta and theta absolute and relative power values compared to children of the same age has been interpreted as a lag in brain maturation [14,16,49].

Group 3 had higher delta and theta AP values than the other groups. The children in this group also had the worst performance in reading, writing and arithmetic subtests. The presence of delta and theta activity in anterior regions agrees with the study by Harmony *et al*. [13], who described the electroencephalographic characteristics of a group of children who took a reading and writing test. In addition to having higher theta values, the group with the worst performance was characterized by the presence of frontal delta activity, which was interpreted as a sign of brain dysfunction. This pattern could explain the deficiencies these children exhibit because frontotemporal regions are not only associated with general attention processes, memory, and executive processes; they are directly related to language processing. Specifically, Brodmann areas 44 and 45, located in the left frontal cortex, are essential to the understanding and production of language as well as to facilitate many other language functions; the inferior, medial, and superior temporal gyri are involved in phonological, semantic, and syntactic processing [50]. Theta values were higher in Group 3 in anterior regions and parietal and occipital areas of the left hemisphere. The word form area, which is systematically activated during reading, lies in the left lateral occipitotemporal sulcus [51]. Posterior language areas are involved in semantic processing as well as phonological processing. Parietal regions are clearly involved in number processing and calculation; the intraparietal sulcus participates in magnitude processing [52] and comparison of symbolic and non-symbolic numerosities [53,54,55]. The angular gyrus is active when subjects perform arithmetic tasks, such as multiplication and sums [56]. The children from Group 3 also exhibit severe deficiencies in arithmetic and calculation processes compared to the other two groups, as seen in the subtests of numerical managing and calculus.

The presence of posterior alpha with adequate reactivity is considered a sign of brain maturity [57,58]. The EEGs of children from Group 3, who showed a worse performance in cognitive tasks than the children of the other two groups, are characterized by a lower alpha AP in the posterior regions. In the visual inspection of the EEG of these children, it can be noted that the dominant rhythm is not alpha; therefore, this lower alpha AP seems to be a consequence of the children not being sufficiently mature for their age, which suggests that their academic difficulties are related to a greater lag in EEG maturation, as has been suggested by John *et al*. [16] and Harmony *et al*. [14].

Beta activity usually does not play a major role at this age [57]. Beta AP decreases with age, although this decrement is not as notorious as the one in delta and theta AP [59,60,61]. Maturation of activity in the beta band progresses from the centre to lateral and, finally, frontal regions [26,60]. Children in Group 3 have significantly more activity in the beta band than children in the other two groups in the frontopolar, anterior temporal and left parietal regions. According to the pattern reported in normal development, these children may have features of immaturity, compared to the other groups with LD, because beta activity has not decreased as much as in the other children in the lateral and frontal areas, which are the final regions to mature. It is noteworthy that the temporal and left parietal regions are involved in language and calculation processes, in which these children have lower performance than other children with LD.

Groups 1 and 2 both had higher AP values in the gamma band frequency range than Group 3; Group 1 was in the range of 30 to 50 Hz and Group 2 in the range of 37 to 47 Hz. Takano and Ogawa [62] report an increase in the resting gamma power that peaks between 4 and 5 years, mostly in the frontal regions, and is then more or less stable, until 12 years, which was the highest age included in the study. The resting state gamma band activity has been linked to the development of better language and cognitive abilities over the first three years of life [63]. Moreover, Gou, Choudhury and Benasich [64] found an association between the gamma power in resting EEG at 16, 24 and 36 months of age and later language, specifically phonological memory and syntactical skills. In this study, higher gamma power was also linked to better performance in the neuropsychological tests. To our knowledge this is the first report of resting gamma activity in children with LDNOS.

On the other hand, it is well known that gamma activity is increased in numerous cognitive processes, such as perception [65], attention and memory [66], expectancy, learning, reading, calculus [67], template matching [68], long term memory formation [69], and language [70]. However, in this work, children were recorded at rest. It is plausible that the higher gamma activity observed in Groups 1 and 2 could correspond to the better performance observed in the neuropsychological tests applied, but this hypothesis should be further evaluated in future research.

As noted above, Groups 1 and 2 have a better cognitive performance and a higher electroencephalographic maturation than Group 3; however, they both have distinct cognitive and electroencephalographic patterns. When looking at the average scores, it would seem that the children in Group 1 do not have low scores; this is because not everyone has low scores in the same domain. What is clear is that Group 1 was characterized by a better reading accuracy and speed as well as better writing accuracy. Accuracy scores are related to the spelling accuracy, or lack of errors, while reading and writing. Group 2 had higher scores in narrative composition, which corresponds to the ability to write a complete story, in a coherent manner, without considering spelling errors. Group 2 has higher values of low alpha power in the bilateral temporal, central and right frontal areas; these higher values of the anterior alpha AP correspond to the presence of frontal activity in the alpha frequency that is reported in children with attention deficit disorder [71] and older adults [72]. It has been hypothesized that this frontal alpha activity is a compensatory phenomenon and thus, it is possible that the children in Group 2 are compensating for some of their functional deficits, such as the higher frontal activity in the delta frequency. In this way, they manage to have similar performance as in Group 1. There were no significant differences between these two groups regarding reading comprehension, writing speed and three arithmetic domains.

On the other hand, Group 1 has more beta2 activity than Group 2 in the frontopolar areas and more gamma1 in the left frontopolar and posterior temporal leads. There is always the possibility that electromyogram is interfering in these frequencies; nevertheless, higher gamma activity in the left temporal areas could explain this group’s better performance in reading and writing accuracy. Shaywitz *et al*. [73] found significant differences between children with dyslexia and a control group in brain activation patterns during a phonological analysis task, observing higher activation in the inferior frontal gyrus, superior temporal sulcus, superior and middle temporal gyri, and medial occipital gyrus in the left hemisphere of healthy children. Accuracy in reading and writing may require a phonological analysis, and the posterior region of the temporal gyrus in the left hemisphere could correspond to the left posterior temporal lead, which is where we are observing higher gamma activity in the group with better performance in this task.

This group of children with LDNOS was very heterogeneous. We identified three subgroups that have a different cognitive profile, and we observed differences in their resting EEG spectral profiles. Moreover, these differences in electrical activity relate to differences in the neuropsychological profile. It is important to consider these differences while planning interventions for children with LD because they could help guide the selection of different strategies in the treatment of children with LD who have different cognitive profiles.
